# A randomized controlled trial evaluating the impact of knowledge translation and exchange strategies

**DOI:** 10.1186/1748-5908-4-61

**Published:** 2009-09-23

**Authors:** Maureen Dobbins, Steven E Hanna, Donna Ciliska, Steve Manske, Roy Cameron, Shawna L Mercer, Linda O'Mara, Kara DeCorby, Paula Robeson

**Affiliations:** 1 School of Nursing, McMaster University, 1200 Main Street West, Hamilton, ON, L8N 3Z5, Canada; 2 Center for Behavioural Research and Program Evaluation, University of Waterloo, 200 University Avenue West, Waterloo, ON, N2L 3G1, Canada; 3 The Guide to Community Preventive Services, National Center for Health Marketing, Centers for Disease Control and Prevention, Atlanta, GA, USA

## Abstract

**Context:**

Significant resources and time are invested in the production of research knowledge. The primary objective of this randomized controlled trial was to evaluate the effectiveness of three knowledge translation and exchange strategies in the incorporation of research evidence into public health policies and programs.

**Methods:**

This trial was conducted with a national sample of public health departments in Canada from 2004 to 2006. The three interventions, implemented over one year in 2005, included access to an online registry of research evidence; tailored messaging; and a knowledge broker. The primary outcome assessed the extent to which research evidence was used in a recent program decision, and the secondary outcome measured the change in the sum of evidence-informed healthy body weight promotion policies or programs being delivered at health departments. Mixed-effects models were used to test the hypotheses.

**Findings:**

One hundred and eight of 141 (77%) health departments participated in this study. No significant effect of the intervention was observed for primary outcome (p < 0.45). However, for public health policies and programs (HPPs), a significant effect of the intervention was observed only for tailored, targeted messages (p < 0.01). The treatment effect was moderated by organizational research culture (*e.g*., value placed on research evidence in decision making).

**Conclusion:**

The results of this study suggest that under certain conditions tailored, targeted messages are more effective than knowledge brokering and access to an online registry of research evidence. Greater emphasis on the identification of organizational factors is needed in order to implement strategies that best meet the needs of individual organizations.

**Trial Registration:**

The trial registration number and title are as follows: ISRCTN35240937 -- Is a knowledge broker more effective than other strategies in promoting evidence-based physical activity and healthy body weight programming?

## Introduction

Currently, there is substantial political and societal pressure to demonstrate the integration of the best available research evidence with local contextual factors, so as to provide the most effective health services in optimizing health outcomes [1]. The purpose of this randomized controlled trial was to evaluate the impact of three knowledge translation and exchange (KTE) strategies in promoting the incorporation of research evidence by public health decision makers into public health policies and programs related to healthy body weight promotion in children.

## Background

### Knowledge translation and exchange: what we know

The integration of research evidence into public health policy and program decision making is commonly referred to as evidence-informed decision making [2], and strategies to promote it as KTE. However, it is well known that the decision-making process is complex, and that multiple forms of knowledge impact both the process as well as the decision. In this study, we were interested in exploring the use of research evidence in decisions concerning the provision of public health services for promoting health body weight in children. In Canada, program managers in public health departments typically make recommendations to senior management on the specific interventions and strategies that could be provided to address particular population issues (*e.g*., healthy weights) [3]. Managers typically explore different options and make decisions about interventions that fit within the social and political climate of their respective regions. We explored whether research evidence influenced these decisions made by program managers concerning whether and which interventions they recommended their health department make available in order to promote healthy body weight in children.

Factors identified previously in the KTE literature known to contribute to clinical and program planning decisions include those related to individual decision makers, the system, patients, and research evidence [4]. At the individual decision-maker level, important factors include past experiences (*e.g*., clinical or managerial experiences with patients/clients, policy makers, events, or circumstances), beliefs, values, and skills; the environment/system level includes resources (both human and financial), legislation, protocols, and societal norms; patient preferences; and research evidence (*e.g*., multiple ways of knowing) [5-8]. The intent of evidence-informed decision making is not to suggest that health policy and program decisions be determined solely by research evidence, but rather research evidence be considered within the context of the setting or circumstance, societal expectations, health care resources, and professional expertise.

Barriers consistently identified to evidence-informed decision making in the KTE literature include: lack of time; limited access to research evidence (*e.g*., many health departments can identify relevant research evidence in the published domain, but experience significant challenges in obtaining the full text in a cost-efficient and timely way) [9,10]; limited capacity to appraise and translate research evidence; and resistance to change (*e.g*., lack of motivation to stop doing what has traditionally been done) [11-17]. System-level changes needed to support evidence-informed decision making include: researchers gaining a better appreciation of the context in which decision makers function and building more collaborative relationships with decision makers [3,18,19].

Three KTE strategies are currently being widely used to promote evidence-informed decision making. These include: freely accessible web-based resources that summarize research evidence; tailored and targeted messages that connect relevant research evidence to specific decision makers [20]; and knowledge brokers (KBs), who work one-on-one with decision makers to facilitate evidence-informed decision making [21]. The internet is established as an essential component of KTE [22], and significant resources have been and continue to be allocated to these strategies. Several web-based resources have been developed with the intent of compiling the best available research evidence by topic area or health care discipline (*e.g*., Medline Plus, More EBN, health-evidence.ca). Some have gone one step further to synthesize the results of the evidence to answer specific practice-based questions [23]. However, there is a scarcity of literature evaluating the effectiveness of web-based resources in achieving evidence-informed decision making.

Tailored and targeted messages have gained momentum as a popular KTE strategy [24-27]. 'Tailored' implies that the message is focused on the specific scope of decision-making authority of the intended user, while 'targeted' indicates that the content of the message is relevant and directly applicable to the decision currently faced by the intended audience. Evidence indicates that computer-tailored messages are associated with increased uptake compared to standardized messages [28], and that electronic targeted messages to subgroups with common interests is effective in promoting evidence-informed decision making [29]. While tailored, targeted messages have been shown to improve uptake of systematic reviews [30], questions remain as to what content is most wanted and required for different audiences, what the most effective communication channels are [28], and which organizations will benefit most from such a KTE strategy.

KBs have been implemented widely in private industry [31-33], and more recently in healthcare settings [21,34,35]. In fact, a great many organizations in Canada have quickly moved to adopt KB roles with little more than anecdotal evidence supporting their effectiveness. A KB acts as a catalyst for systems change, establishing and nurturing connections between researchers and end users [36], and facilitating learning and exchange of knowledge [37]. The anecdotal evidence suggests that KBs improve the quality and usefulness of evidence that is employed in decision making [38], while promoting a decision-making culture that values the use of evidence [39,40]. Furthermore, the heightened degree of interaction with decision makers through knowledge brokering is assumed by many to be the optimal KTE strategy in comparison to less interactive strategies; however, this has yet to be proven [34]. Given the lack of evaluation of each of these KTE strategies individually or in comparison to one another, the timing was right for conducting this study.

### Healthy body weight

The problems of obesity, overweight, and physical inactivity have been identified in children [41]. According to the latest Canadian Fitness and Lifestyle Research Institute Physical Activity Monitor [42], 90% of Canadian children and youth aged five to 17 are not active enough to promote good health. Many of the risks associated with obesity in children cluster in cardiovascular disease risk factors known as the insulin resistance syndrome, and have been identified in children as young as five years of age [43]. In addition, overweight in childhood increases the risk of death from ischemic heart disease in adulthood two-fold over 57 years, and the incidence of Type 2 diabetes is increasing and is attributable to obesity [44]. Most alarming, however, is the knowledge that physical activity patterns and chronic disease conditions track from childhood into adulthood [45-55]. Canadian research estimates that physical inactivity and obesity resulted in expenditures of $5.3 and $4.3 billion in direct and indirect costs, representing 2.6% and 2.2%, respectively, of total health care costs in Canada [56].

The literature demonstrates that regular aerobic activity increases exercise capacity and plays a role in both the primary and secondary prevention of cardiovascular disease [57-60]. Furthermore, regular physical activity has been shown to enhance health, reduce the risk for all-cause mortality, prolong life, and improve quality of life [61-67]. The evidence suggests that the best primary strategy for improving the long-term health of children and adolescents may be in creating a lifestyle pattern of regular physical activity and healthy eating that will carry over to the adult years [68].

### Promoting healthy body weight in children: the role of public health

Public health departments in Canada are responsible for promoting the health of the population, preventing disease, and providing medical care to treat communicable diseases. They provide services that focus on promoting the health of individuals as well as health promotion within schools and worksites, nutritional counseling, physical activity promotion, development of community strengths to promote/improve health, and the promotion of healthy environments [69]. The public health sector in Canada is structured generally with a medical officer of Health at the head of the organization and who has senior decision-making authority (subsequent to the local/regional board of health) for the services provided by that organization to a designated local community or region. The public health workforce responsible for the promotion of physical activity and chronic disease prevention is comprised primarily of public health nurses, nutritionists, physical activity experts, and health promotion officers. At the time this study was conducted (July 2004 to February 2006), all provinces and territories in Canada held mandates requiring public health departments to develop and implement strategies to promote healthy body weight in children. Despite these mandates, there was limited capacity (time, skill, access) among public health decision makers, and limited resources to utilize the best available research evidence with which to plan and implement effective healthy body weight promotion programs and services.

## Methods

### Design

**Figure 1:**
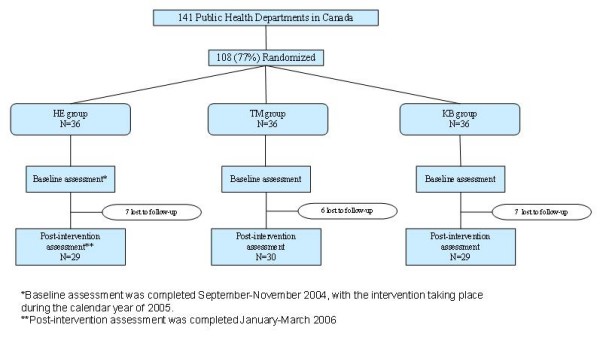
**Flow chart of data collection from baseline to post intervention**. Flow chart showing the process of data collection from baseline to post intervention.

This randomized controlled trial funded in 2003 by the Canadian Institutes of Health Research, was the first in Canada to evaluate the effectiveness of a KB in comparison to other KTE interventions on promoting evidence-informed decision making in public health departments. Following ethics approval (McMaster University Faculty of Health Sciences Research Ethics Board) and recruitment, participating health departments were stratified according to size of population served and randomly allocated to groups using computer-generated random numbers. Given the background work conducted by the research team, as well as findings from the literature, stratifying public health departments by size of population served prior to randomization was deemed necessary. The three strata were defined as: health departments serving a population size below 50,000; a population size between 50,000 and 250,000; and a population size above 250,000. The Statistics Canada Peer Groups were used to allocate public health departments to each strata. The public health departments were randomly allocated to intervention groups in equal numbers within strata by computer generated pseudorandom draws using standard algorithms. Three health departments that remained unselected after equal allocation within strata were assigned to treatment groups randomly across strata. The health department was the unit of analysis. The study process is shown in Figure 1.

The framework proposed by Dobbins *et al*. [70] is one of many frameworks [71-76] that have been developed to illustrate the process of knowledge translation and evidence-informed decision making. Dobbins' framework was used to guide the development of the KTE strategies (tailored, targeted messages and KB) and identify relevant outcomes for this study. The framework demonstrates the complex inter-relationships that exist between the five stages of innovation identified by Rogers, [77] (knowledge, persuasion, decision, implementation, and confirmation), and four types of characteristics, organizational, environmental, individual, and the innovation [78], as the knowledge translation process occurs. The framework also identifies the variety of possible outcomes that can be observed including: knowledge and attitudes; decision making; implementation (*e.g*., putting research knowledge into public health policy and practice, guideline development); and outcomes (*e.g*., changes in public health policy and practice). This study focused on the measurement of outcomes, specifically changes in public health policies and programs at the local public health department level.

The hypotheses were: public health departments exposed to tailored, targeted messages and the KB would report greater evidence-informed decision making than those exposed to a repository of quality assessed systematic reviews evaluating public health interventions (health-evidence.ca); knowledge brokering would result in greater evidence-informed decision making than tailored, targeted messages; and characteristics of the organization would have significant impacts on the effect of the KTE interventions on evidence-informed decision making. More specifically, we hypothesized that certain organizational characteristics (*e.g*., research culture, or the value organizations placed on the use of research evidence in decision making) would have an impact on the effectiveness of the KTE interventions to promote evidence-informed decision making. A previous study with Canadian public health decision makers illustrated that public health departments that valued the use of research evidence in decision making were significantly more likely to use research evidence for program planning decisions than health departments that put less value on research evidence [79,80]. Therefore, we hypothesized that organizations that placed lower value on using research evidence in decision making would experience less improvement in evidence-informed decision making than those who valued research evidence more highly.

### Sample and recruitment

The sample was comprised of regional and local public health departments in Canada. Eligible participants from participating health departments were directly responsible for making program decisions related to healthy body weight promotion in children. This included program managers and/or coordinators in Ontario, and program directors in the rest of Canada. All health departments in Canada were invited to participate. Health departments in Canada were identified through provincial databases. Participants were recruited into the study in a two-stage process. First, consent from the most senior person in the public health department (*e.g*., medical officer of health or chief executive officer) was sought. If written consent was obtained, the name of the person most directly responsible for making decisions related to healthy body weight promotion among children was identified and contacted. A letter of invitation was then sent directly to the potential participant followed by a telephone call to ascertain consent to participate in the study and answer any questions.

### Intervention

The three interventions were implemented simultaneously during 2005. The content used in the KTE interventions (healthy body weight promotion in children) was summarized from seven rigorous systematic reviews and will be described in greater detail in the outcomes section. The least interactive KTE intervention was access to health-evidence.ca (HE group). Health-evidence.ca is a repository of all systematic reviews published since 1985 evaluating any public health intervention. All participants in the study received electronic communication about the availability of this site. Upon searching this site for reviews evaluating strategies to promote healthy body weight in children (to mimic the standard way in which electronic sources are utilized in practice), those in the HE group would have become aware of the title, citation, and assessment of the methodological quality of seven systematic reviews evaluating the effectiveness of interventions to promote healthy body weight in children. Participants in the HE group also had access to the published abstracts, and the full text articles (copyright purchased for this study) through Health-evidence.ca. Finally, a short summary for each of the systematic reviews, written by the research team, identified the key findings and recommendations for public health policy and practice that were directly applicable to the types of decisions for which the participants were responsible. Such summaries are written for all of the well-done systematic reviews appearing in health-evidence.ca and are available to all users, while targeted primarily at the level of program managers.

The moderately interactive KTE intervention included tailored, targeted messages plus access to health-evidence.ca (TM group). The TM intervention included sending participants a series of emails that included the title of the seven systematic reviews followed by a link to the full reference, including abstracts, on health-evidence.ca. The online reference offered a link to the short summaries, and finally, the full text of each review. Over seven successive weeks, on the same day each week and the same time of day, participants in the TM group were sent an email indicating that a systematic review related to healthy body weight promotion in children was available in full text at the link provided. At the URL linked within the email message, participants also received access to the PDF version of the systematic review, the published abstract of the review, as well as the short summary written. Finally, the text of the message was worded to say, 'this message is number XX in a series of seven emails you will receive on healthy body weight promotion in children as part of the KTE strategy you are being exposed to in this randomized controlled trial'.

The most interactive KTE intervention included both the HE and TM components and a KB who worked one on one with decision makers in the public health departments. One full-time KB provided knowledge brokering services to all English speaking participants allocated to the KB group (n = 30). A second Francophone KB (0.2 full time equivalent) provided KB services to French-speaking participants also allocated to the KB group (n = 6). The KBs were Master's prepared, had extensive knowledge and expertise in public health decision making, as well as an understanding of the research process. Specific tasks conducted by the KB included: ensuring relevant research evidence related to healthy body weight promotion was transferred to the public health decision makers in ways that were most useful to them, assisting them to develop the skill and capacity for evidence-informed decision making, and assisting them in translating evidence into local practice.

Approximately twenty percent of KB time was spent facilitating knowledge and skill development either through face-to-face interaction such as workshops or online strategies such as webinars, interactive web-enabled meetings, or conferences. Eighty percent of the brokers' time was spent preparing for and directly interacting with participants. The proportion of time the KB spent preparing for interaction with participants was 40% to 50% early in the project and declined to 30% as both public health decision makers and the KB became more skilled in their respective roles. KB activities were classified into the following categories: initial and ongoing needs assessments; scanning the horizon; knowledge management; KTE; network development, maintenance, and facilitation; facilitation of individual capacity development in evidence-informed decision making; and facilitation of and support for organizational change. These activities were carried out through regular electronic and telephone communication, and one site visit to each health department of one to two days in length. As well, each health department was invited to attend a one-day workshop held regionally (four cities) across Canada. A more complete description of the KB intervention is published elsewhere [81]. However, the main activities of the KB intervention are described.

At the start of the intervention, the KB conducted assessments at the individual, organizational, and environmental levels, in order to identify strengths, knowledge, and capacity for evidence-informed decision making. The KB then worked with participants to generate a plan for developing individual and organizational capacity for evidence-informed decision making. In order to facilitate participant access to the best available evidence, the KB consistently scanned the horizon for new evidence and resources of interest to participants. This activity involved maintaining subscriptions to related list serves, electronic distribution lists, and e-table of contents alerts from relevant journals. The majority of the KB's time was spent doing KTE, which was facilitated by developing and maintaining a trusting relationship with participants. The KB-initiated communication with participants occurred at a minimum of once per month, and more frequently as requested. The KB also offered a site visit to each public health department. The purpose of the site visit was to facilitate the building of a trusting relationship between the health department and the KB, as well as to enable the KB to learn more about the local context. This facilitated the tailoring of KB services to the specific needs of each local environment. Furthermore, the activities conducted by the KB during each site visit varied according to specific needs and goals identified by each health department. In many cases, the KB participated in team program planning sessions and assisted in the interpretation of evidence from the tailored, targeted messages and its incorporation into local program plans. The KB also conducted training sessions in many health departments to assist participants and their colleagues in developing their capacity to be critical consumers of different knowledge sources. Opportunities to facilitate knowledge, skills development, and capacity for evidence-informed decision making occurred during all interactions with the KB at the individual (email, telephone, site visit) and group level (site visit, regional workshop, webinars). Finally, during the regional workshops, the KB presented the results of the systematic reviews to participants, facilitated discussion concerning the results as well as implications for local program and public health policy development. KBs also encouraged participants to engage in individual and joint problem-solving related to evidence-informed decision making, and enabled face-to-face contact with the KB to promote credibility and trust.

### Data collection

The data were collected using a telephone-administered survey (knowledge transfer and exchange data collection tool) at baseline (August 2004) and immediately post-intervention (February 2006). Items in the questionnaire were chosen from questionnaires previously tested and used in diffusion of innovation and research utilization studies [11,77,82-88]. We tested the modified questionnaire for reliability and validity among public health decision makers, and have reported a Cronbach alpha of 0.65 for reliability elsewhere [11,80,89]. The questionnaire is available from the corresponding author upon request. The questionnaire was administered twice to participants at baseline, one month apart.

### Independent variables

Data were collected on organizational, environmental, and individual characteristics shown previously to be related to evidence-informed decision making [79], and measured using seven-point Likert scales. Organizational characteristics included: organizational culture (*e.g*., research culture, or the value placed on using research evidence in decision making, and the expectation to demonstrate use of research evidence in decision making), staff training in research methods and critical appraisal, and decision-making style. The environmental characteristic included collaboration with other community organizations. Individual characteristics included age, education, position, perceived influence over the decision-making process, and perception of the barriers to using research evidence in public health decision making. All variables were measured in the same direction.

### Dependent variables

Two dependent variables were evaluated: global evidence-informed decision making and public health policies and programs. For global evidence-informed decision making, participants were asked to report on the extent to which research evidence was considered in a recent program-planning decision (previous 12 months) related to healthy body weight promotion. This is a common way of measuring research use in the KTE field. Participants were asked to quantify their response ranging from one (not at all) to seven (completely). However, given many have suggested that this is not an optimal way of measuring evidence-informed decision making, we developed a second outcome variable, labeled 'public health policies and programs'. This measure was derived as the sum of actual strategies, policies, and/or interventions for healthy body weight promotion in children being implemented by the health department.

Eleven policies, programs, and/or interventions with good evidence of effectiveness were identified from seven systematic reviews assessed as being of high methodological quality [90-96] (Table 1). Each systematic review was assessed for methodological quality by two independent reviewers using a previously developed and tested quality assessment tool [97,98]. Reviewers met to discuss ratings, and consensus on all ratings was achieved. Only those systematic reviews attaining seven points or higher out of a total of ten possible points were deemed of sufficient methodological quality to inform public health policy and practice. Participants were asked whether the public health policies and programs were being implemented by their health department (yes/no). The total number was summed and compared across groups from baseline to post intervention.

**Table 1:** Healthy body weight policies and programs (HPPs)

Recommended Intervention/Program/Policy	Supporting Systematic Review Evidence
Interventions are focused on changing behaviour as opposed to gaining knowledge	Ciliska (2000) [91], Dishman (1996) [92], Kahn (2002) [94], Thomas (2004) [96]

Interventions are multi-component and targeted at changing behaviour	Campbell (2002) [90], Ciliska (2000) [91], Hardeman (2000) [93], Thomas (2004) [96]

Interventions include messages targeted at specific behaviours (*e*.*g*., increased fruit and vegetable consumption)	Ciliska (2000) [91], Thomas (2004) [96]

Interventions target high risk populations	Hardeman (2000) [93]

Interventions include a goal setting component for individuals	Kahn (2002) [94], Thomas (2004) [96]

Interventions include the use of small groups	Dishman (1996) [92], Kahn (2002) [94]

Interventions include messages targeted at decreasing sedentary behaviour and increasing physical activity	Campbell (2002) [90], Dishman (1996) [92], Kahn (2002) [94]

Interventions advocate for an increase in the number of physical activity classes required during school hours	Campbell (2002) [90], Kahn (2002) [94]

Interventions advocate for an increase in the amount of aerobic activity provided during school hours	Kahn (2002) [94], Thomas (2004) [96]

Interventions advocate for regular classroom teachers to receive training and mentoring from specialists OR for specialists to teach physical education classes	Campbell (2002) [90], Ciliska (2000) [91], Thomas (2004) [96]

Interventions promote family and/or community involvement	Dishman (1996) [92], Kahn (2002) [94]

### Analysis

Mixed-effects models were used to conduct tests of the two hypotheses related to the treatment effects, which is a standard approach to the analysis of designs with repeated measurements [99]. Repeated measurements over time were modeled as nested within participants, and time of observation was coded to estimate the differences between groups in scores at the average of the two baseline observations, and then the change from baseline to the post-intervention follow-up. The interaction of this change with the randomized treatment assignment is the appropriate estimate of the treatment effect, such that we tested whether change following the intervention differs among the intervention groups. These mixed-effects models provide for appropriate adjustment for the repeated measurements with participants when testing treatment effects, and they also allow for flexible handling of missing data. The moderating roles of selected predictor characteristics (hypothesis three) were also tested by evaluating their three-way interactions with time and treatment.

## Results

All 141 public health departments in Canada were invited to participate in this study, of which 108 (77%) agreed to do so. Stated reasons for not participating included undergoing restructuring, involved in too many research studies, or the topic was not a priority. Thirty-six public health departments were assigned to each of the three intervention groups. No statistically significant differences were observed between groups at baseline on important independent and dependent variables.

### Follow-up data

Participation by province and territory ranged from 29% to 100% with the sample consisting primarily of health departments serving both urban and rural populations (46%). Table 2 presents a description of the study sample. Follow-up data were collected from 88 of 108 (81.5%) participating public health departments. Reasons for not participating in the follow-up survey were lack of time and not having anyone working in healthy body weight promotion. Among the HE, TM, and KB groups, similar drop-out rates were observed of seven, six, and seven health departments, respectively.

**Table 2:** Baseline characteristics of public health departments and decision-makers

Characteristic	Total Sample (means)
Positions:	
Front line staff	35%
Managers	26%
Directors	10%
Coordinators	9%
Other	20%

Discipline:	
Nurse	47%
Nutritionist	19%
Physical education specialist	4%
Physician	2%
Other	26%

Years in current position	5

Years in public health	13

Frequently hear the terms research or research evidence.	5.4*

My organization highly values the use of research evidence in decision making.	5.2*

My supervisor expects me to use research evidence in program planning decisions.	5.6*

Research evidence is consistently used in program planning decision making.	4.9*

I have access to someone who can help me interpret and apply research evidence.	4.5*

The health unit's governing board is influenced by research evidence.	4.8*

How helpful is research evidence to you for program planning decisions?	5.4*

Is it easy to access relevant research?	4.8*

You find policies/programs described as effective in the literature are affordable in practice.	3.7*

Research in your field is done with populations similar to the populations you serve.	3.9*

Have you ever seen a systematic review relevant to your field?	79% responded yes

How would you rate the availability of systematic reviews relevant to your field?	4.1*

How would you rate systematic reviews you are familiar with for ease of use?	4.8*

* based on a seven-point scale

### Intervention integrity

It is unknown to what extent the HE group accessed http://www.health-evidence.ca. To our knowledge, all those exposed to the TM intervention received 100% of the intervention. For those exposed to the KB intervention, approximately 70% received the full intervention (*e.g*., frequency, intensity) with approximately 15%, respectively, not engaging at all, or to a limited extent. Organizations were analyzed according to their assigned group.

### Outcomes

The estimates from the mixed-effects models are presented in Table 3. The table gives estimated pair-wise differences for the TM and KB groups, relative to control (HE group), as well as overall tests of group differences at baseline and for the change from baseline to follow-up. In addition, the standard deviation in outcome between and within health departments over time is provided. This gives an indication of the degree of variation in the outcomes that remains unexplained after accounting for the intervention. For both outcomes, most of the remaining variation appears as unexplained changes over time within health departments. Table 3 shows that baseline scores do not differ significantly between groups for either outcome, although the TM group possibly had fewer public health policies and programs at baseline compared to the HE group (p < 0.06).

**Figure 2:**
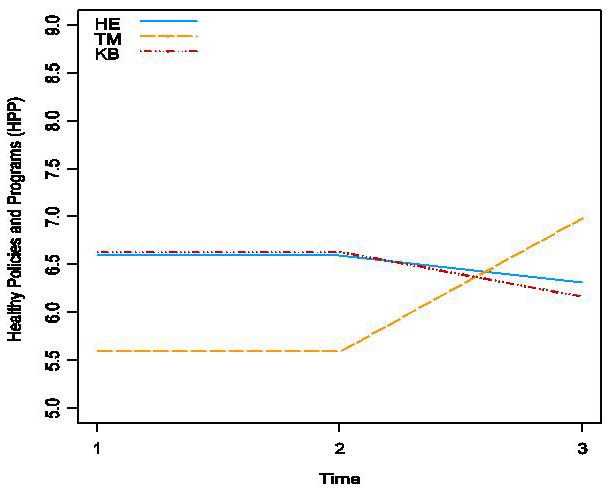
**Framework for Research Dissemination and Utilization**. This figure depicts the primary author's framework for research dissemination and utilization.

**Figure 3:**
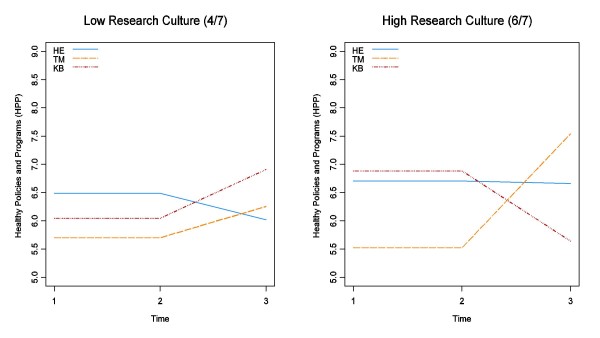
**A. Comparison of Health Policies and Programs (HPP) Post-Intervention**. This figure shows the association between the interventions (control, tailored messaging, and knowledge brokering) and the number of evidence-supported health policies and programs (HPP) post-intervention. B: *Impact of Low versus High Organizational Research Culture on Health Policies and Programs*.

As shown in Table 3, the intervention had no significant effect on global evidence-informed decision making (p < 0.45), although all groups improved to some extent. For public health policies and programs, as is shown in Figure 2, a significant effect of the intervention was observed (p < 0.01). For this outcome, the TM group improved significantly from baseline to follow-up in comparison to the HE and KB groups that showed no significant change. With respect to hypothesis three, some organizational characteristics were shown to moderate the intervention effect, although not always in the hypothesized direction. When organizational research culture was added to the mixed-effects models as a predictor, the group/time/culture interaction was significant (p < 0.03). This three-way interaction is illustrated in Figure 3, with the predictions for each group shown at relatively low (four of seven) and high (six of seven) values of the extent to which health departments reported they valued research evidence.

**Table 3:** EIDM outcomes baseline to follow-up

	Global EIDM		HPP	
	
	Estimate (95% CI)	overall p-value	estimate (95% CI)	overall p-value
**Baseline in HE**	5.43 (5.11;5.75)		6.50 (5.91,7.28)	
**versus TM**	0.18 (-0.30;0.66)	p < 0.73	-1.01 (-1.98,-0.03)	p < 0.06
**versus KB**	0.02 (-0.44;0.48)		0.03 (-0.95,1.02)	

**Post-Tx change in HE**	0.74 (0.26;1.22)		-0.28 (-1.20,0.65)	
**versus TM**	-0.42 (-1.10;0.26)	p < 0.45	1.67 (0.37,2.97)	p < 0.01
**versus KB**	-0.09 (-0.78;0.60)		-0.19 (-1.50,1.12)	

**Between-health department SD**	0.53 (0.35;0.81)		1.38 (1.05,1.81)	
**Residual SD**	0.94 (0.82;1.07)		2.06 (1.85,2.29)	

As Figure 3 illustrates for health departments with low organizational research culture, the intervention effect was as we hypothesized -- the control group was unchanged, the TM group improved somewhat, and the KB group improved most. However, when organizational research culture was high (six on a seven-point scale), the HE group remained unchanged, the KB group decreased (fewer public health policies and programs), and the TM group increased significantly. Similar trends were observed for organizational characteristics, such as expectation to use research evidence and frequency of hearing the term research evidence. However, no significant differences in results were observed when multiple organizational characteristics were included in the models, therefore only the results of organizational research culture have been presented.

## Discussion

Generally the results of this randomized controlled trial show the need to match the organizational research culture to intervention type, and in particular support the hypothesis that tailored, targeted messages plus website informational materials can be an effective strategy for facilitating evidence-informed decision making. The results indicate that the 'right' evidence, 'pushed' out to the right decision maker working in an organization supportive of evidence-informed decision making, leads to outcomes in the hypothesized direction. In addition, simply having access to an online registry of research evidence appears to have no impact on evidence-informed decision making. Finally, knowledge brokering does not appear to be effective in promoting evidence-informed decision making overall, although there appears to be a trend toward a positive effect when organizational research culture is perceived as low.

These findings are supported by published studies showing that simple KTE interventions can be as effective as complex, multi-faceted ones [100-102]. A recent meta-analysis evaluating the effectiveness of KTE strategies found that reminders resulted in improved uptake of research evidence compared to more complex, multi-faceted KTE strategies [103]. It might be that complex, multi-faceted interventions dilute the key messages of the intervention making it difficult for decision makers to know what they should do.

As is depicted in Figure 2, that TM is optimal to both HE and KB interventions, it may be that TM provides decision makers with just the 'right amount' of information that has direct relevance to their practice, thereby making it easier to incorporate the evidence into program planning decisions. These results are supported by Hawkins *et al*., who found that TM employs strategies of personalization, feedback, and content matching, and that these factors work together to facilitate research use [104]. In our study, the TM intervention employed personalization and content matching, given that each decision maker received individualized messages directly matched to their current area of decision-making authority. The results suggest that passive KTE strategies, such as access to high quality synthesized evidence that the HE group had access to, is insufficient to facilitate evidence-informed decision making. It implies that in order for KTE strategies to be effective, the evidence (*e.g*., evidence that is relevant, high quality and synthesized) must be actively delivered directly to decision makers, rather than requiring decision makers to access it themselves, even if it is in one place. Furthermore, the results depicted in Figure 2 also imply that TM is optimal in comparison to knowledge brokering. It may be that the brokering intervention implemented in this study sought to address multiple aspects of the process of evidence-informed decision making, namely questioning practice, turning practice-based issues into answerable, searchable research questions, and being a critical consumer of all forms of evidence. It may be that greater attention was paid to developing skill and capacity in these areas, which may have slowed down the process of decision making during the period of follow-up. This may partially explain why the KB intervention in Figure 2 appears to have had no impact on evidence-informed decision making from baseline to follow-up or in comparison to the TM intervention.

The results also suggest that TM is only effective for certain organizations. As is depicted in Figure 3, the extent to which the organization valued research evidence in decision making affected the impact of the TM intervention. For example in Figure 3a where research culture was perceived as relatively low, the TM group only benefited slightly from the TM intervention. In comparison however, the TM group improved greatly when the research culture was rated as high, as is shown in Figure 3b. It may be that those health departments reporting a high research culture are already motivated to use research evidence and what they require most is facilitated access to rigorous, summarized, relevant research evidence personalized to their decision making needs, in order to achieve evidence-informed decision making. McGregor *et al*. [105] reported similar findings that policy-makers were more likely to use the results of technology assessments when they requested information, and when they received the information while still engaged in making a decision on that topic. Furthermore, the results suggest that TM is not sufficient for public health departments with low research culture. It may be that in organizations with low research culture, there are other barriers to using research evidence in decision making, and that facilitating access to the research evidence does not overcome these challenges. It implies that other strategies may need to be employed to overcome barriers to evidence-informed decision making, prior to implementing a TM strategy.

One might question why organizations with high organizational research culture require tailored, targeted messages. One explanation might be that decision makers face incredible barriers to evidence-informed decision making, most notably time to find, retrieve and translate research evidence. Therefore, KTE strategies that minimize the time barrier and that assist the process of translation by tailoring and targeting findings to decision maker needs, intuitively make sense.

The inability to demonstrate a positive effect of the KB intervention counters widely held assumptions that a customized, highly interactive KTE strategy results in greater evidence-informed decision making [106]. Some have suggested that knowledge brokering is a long and involved process [107-109]. It addition to the explanations provided earlier, it is also possible that the duration and intensity of the KB intervention was insufficient to facilitate significant changes in evidence-informed decision making. It is also possible, as suggested recently [110,111], that facilitation of a community of practice was an important element missing from our KB intervention. Our KB also did not have access to a network of KBs for guidance and support, which has been shown to be crucial for optimal implementation of similar roles [112-114].

An interesting finding was the moderating effect research culture had on public health policies and programs for those in the KB group. For example, as Figure 3a illustrates, health departments with perceived low research culture exposed to the KB intervention experienced a significant and positive improvement in the number of public health policies and programs. However, no benefit and possibly a decrease in public health policies and programs was observed when research culture was high. This suggests that a KB may be effective for certain health departments and not others, particularly those not engaged in evidence-informed decision making. It is possible that KBs facilitate the development of capacity and support in health departments with low research culture, which may be an important precursor to evidence-informed decision making. Cillo [115] reported similar results, suggesting a KB had greater success when the role was matched well to both organizational context and the complexity of market knowledge. The findings demonstrate the importance of assessing characteristics of each organization (*e.g*., research culture), and then using this information to tailor KTE strategies to meet the needs of each organization. Furthermore, it may be that those organizations exposed to the KB intervention that had high research culture at baseline did not perceive the KB intervention as useful and did not engage in the intervention, or it may be that exposure to the KB resulted in some health departments revisiting their efforts towards evidence-informed decision making, and in doing so, re-evaluated recent policy and program decisions.

### Outcome measurement

Our primary outcome, Global evidence-informed decision making, may not be optimal for measuring KTE effectiveness despite its consistent use in the literature. It is likely that this measure is too vague to elicit reliable and valid responses. One conclusion is that concrete outcome measures, such as public health policies and programs that are tied to specific behaviors and/or programs, may provide a more concrete measure of evidence-informed decision making. However, challenges still exist with the use of this measure because the existence of organizational policies does not necessarily translate into actual services being provided, and it is unclear what the optimal data source is for identifying public health policies and programs that are being implemented. Priorities for future research include: development and testing of data collection tools for measuring more objectively evidence-informed decision making outcomes; and continued exploration of subjective measures so as to better understand evidence-informed decision making and KB processes, as well as indicators of KB success.

### Limitations

The limitations in this study include: the source of data, participant turnover, exposure to the intervention, and self-reported outcome measures. A decision to have just one participant from each organization provide data was made following an assessment of Canadian public health departments that suggested services for healthy body weight promotion in children were coordinated across organizations. In reality, these programs span multiple divisions (*e.g*., healthy lifestyles, family health) and multiple teams within divisions (*e.g*., nutrition, physical activity, schools), resulting in many public health professionals working simultaneously on different interventions, programs, and policies, usually with limited knowledge of what others are doing. It is possible that the participants in our study had inadequate knowledge to accurately report on all public health policies and programs provided by their organization. This may have led to both under- and over-reporting of this outcome. One strategy to overcome this issue would be to have multiple participants from each health department participate in data collection and report only on those interventions, programs, or policies directly relevant to themselves. Furthermore, while the KB encouraged multiple decision makers from each public health department to participate in the KB intervention, for many health departments only one decision maker was exposed to the intervention. This likely resulted in insufficient exposure to the intervention among health department staff to facilitate change at the organizational level.

A significant limitation of this study was high participant turnover. While the majority of health departments (81.5%) completed the study, different decision makers completed the baseline and follow-up surveys in 30% of health departments. This reflects the transient nature of public health in Canada and in the United States, and is not something that could have been avoided. This high turnover rate may have resulted in substantial error in outcome measurement and may explain some of the huge variation in the number of public health policies and programs observed from baseline to follow-up in some health departments. This continues to represent a significant issue, and one that we are unable to quantify in terms of its impact and in which direction on the data. Furthermore, given that up to 30% of participants either did not engage with the KB at all or to a limited extent, it is possible that the results of this study are generalizable only to those health departments that would engage with the KB.

The challenges we encountered in conducting this randomized controlled trial raise issues concerning the appropriateness of using empirical designs in evaluating the effectiveness of KTE strategies, particularly in public health settings. Of particular importance is the inability, through randomization, to eliminate all differences (particularly organizational ones) between comparison groups other than the intervention. This poses difficulties with interpretation of the results as it is unclear whether findings truly represent what actually occurred, or if inherent differences in organizational context masked or moderated the treatment effect. While the findings of this study (TM is effective only for organizations with certain characteristics) contribute important knowledge to the field, additional research is needed to better understand the how, what, where, and when with respect to the effectiveness of the KTE strategies. Future research in this field should integrate other designs to better understand in which circumstances KTE strategies work, for whom, and why [116]. Other designs likely to be useful and used extensively in the business literature are case studies, interrupted time series, 'N of 1' studies, and qualitative studies such as grounded theory. Mixed methods designs will allows us to understand more fully not only if KTE strategies are effective, but also more importantly why or why not, how and why they work, what impedes impact, and when KTE strategies will have the greatest likelihood of having a significant and positive impact. Finally, additional research is required to develop and test a tool for assessing organizational factors associated with evidence-informed decision making. While some assessment tools exist, no one tool currently stands out as being optimal.

## Conclusion

The results of this study suggest that tailored, targeted messages are more effective than a KB or access to http://www.health-evidence.ca, particularly in organizations with a culture that highly values research. Lessons learned suggest a greater emphasis on the identification of organizational characteristics so as to identify and implement an optimal array of KTE strategies, that more attention to appropriate outcome measures is needed, and that alternative research designs may be necessary in really understanding KTE impact.

## Competing interests

The authors declare that they have no competing interests.

## Authors' contributions

MD conceived of the study, participated in the analysis and drafted the manuscript. PR provided the intervention and assisted in draft of the manuscript. DC, SH, RC, LO, KD, SM, and SH consulted on the intervention as it was designed and provided, and participated in review of the manuscript. All authors read and approved the final manuscript.
